# Prevalence of Children’s Mental Health Problems and the Effectiveness of Population-Level Family Interventions

**DOI:** 10.2188/jea.JE20140198

**Published:** 2015-08-05

**Authors:** Noriko Kato, Toshihiko Yanagawa, Takeo Fujiwara, Alina Morawska

**Affiliations:** 1 Area on Health Promotion Research, National Institute of Public Health, Wako, Saitama, Japan; 1 国立保健医療科学院 地域保健システム研究分野 (現十文字学園女子大学); 2 School of Health and Nursing Science, Wakayama Medical University, Wakayama, Japan; 2 和歌山県立医科大学 保健看護学部; 3 Department of Social Medicine, National Research Institute for Child Health and Development, Tokyo, Japan; 3 国立成育医療研究センター 社会医学研究部; 4 Parenting and Family Support Centre, The University of Queensland, Queensland, Australia; 4 クイーンスランド大学 家族子育て支援研究所

**Keywords:** child, mental health, prevalence, family intervention, evaluation

## Abstract

The prevalence of mental health problems among children and adolescents is of growing importance. Intervening in children’s mental health early in life has been shown to be more effective than trying to resolve these problems when children are older. With respect to prevention activities in community settings, the prevalence of problems should be estimated, and the required level of services should be delivered. The prevalence of children’s mental health disorders has been reported for many countries. Preventive intervention has emphasized optimizing the environment. Because parents are the primary influence on their children’s development, considerable attention has been placed on the development of parent training to strengthen parenting skills. However, a public-health approach is necessary to confirm that the benefits of parent-training interventions lead to an impact at the societal level. This literature review clarifies that the prevalence of mental health problems is measured at the national level in many countries and that population-level parenting interventions can lower the prevalence of mental health problems among children in the community.

## Abstract (ja)

小児及び思春期におけるメンタルヘルス問題の有病割合は極めて重要性が増している指標である。子どものメンタルヘルスに早くから介入することは、子どもがより年齢が大きくなってからの介入より有効である。地域レベルでの予防活動においては、健康課題の有病割合を把握した上で、必要なレベルのサービスを提供する必要がある。小児のメンタルヘルス有病割合は多くの国で明らかにされている。予防活動においては、環境を最善にすることが強調されている。親は子供の成長に大きな影響を及ぼすことから、育児技術を向上させるための親教育に大きな関心が注がれている。そのなかで、親支援介入の地域レベルでの効果を明らかにする公衆衛生的アプローチが求められる。本文献レビューによって、小児メンタルヘルスの有病割合が国レベルで把握されている事例が多く、地域レベルでの親支援介入が地域での小児メンタルヘルス問題の有病割合を減少させうることが明らかになった。

## INTRODUCTION

Within the last century, considerable change has been observed in the health and disease patterns of children and young people.^1^ One feature of this “millennial morbidity”^2^ is the growing importance of mental health problems. For example, the World Health Organization (WHO) has predicted that internalizing disorders will surpass those of HIV/AIDS in terms of disease burden by the year 2030.^3^ Further, emotional and behavioral problems have become increasingly common among children.

Mental health problems can be a major burden on individuals in everyday situations, such as social relations with friends, family happiness, and school functioning. In addition, mental health problems can be very long-lasting.^4^ If childhood problems are left untreated, only approximately 50% of preschool children show a natural reduction in behavioral problems. The remaining 50%, however, may experience long-term sequelae, including serious consequences such as a breakdown of family functioning and dropping out of school. Further, alcohol and drug abuse may occur as a result of the development of depression during adolescence and adulthood. This situation imposes a large cumulative drain on society by impairing productivity and incurring social and financial costs associated with sub-optimal participation in the labor force and failure to utilize clinical treatment services.^5^

Interventions that occur earlier in one’s life have been shown to be preferable to those occurring later in life, in terms of cost and effectiveness.^6^ Therefore, preventive strategies are essential to ensure that problems are dealt with early. Preventive interventions have emphasized optimization of the environment to prevent or manage children’s behavior. Because parents are the primary influence on their children’s development, considerable attention has been placed on the development of parent training to strengthen parenting skills to prevent the onset of behavioral difficulties.^7^ There is clear evidence linking poor parenting and family risk factors to worsening of behavioral problems. The main purpose of parenting programs is to develop parents’ ability to observe, identify, and respond to their children’s behaviors in new, more effective ways.

Parent-training programs have been developed as one component of comprehensive prevention and intervention methods for families of children with behavioral problems.^8^ Clinical trials have suggested that parent training improves parents’ child-management skills and reduces children’s misbehavior.^9^^–^^18^ In addition, parenting interventions lead to increased parent confidence, reduced stress, and improved family relationships.^17^

Parenting programs have great potential to improve children’s quality of life, mental health, and family relationships, and to benefit the general public. However, traditional clinical models of service delivery cover a relatively small number of parents. A public-health approach is necessary to reach a larger number of parents and to have a societal-level impact.^15^ While clinic-based parent-training trials have been shown to be effective for families who visit the clinic, the proportion of parents who are not referred and have a need for these services is not known. To avoid biases that result from clinic-based studies, obtain a more representative community sample, and estimate the percentage of high-risk families who need parent training, a community approach that screens all kindergartens and/or schools in the community and identifies children who have behavioral problems is needed.

In order to develop effective prevention approaches for children’s mental health problems in community settings, it is essential that good estimates of the prevalence of such problems are available in order to plan and deliver appropriate services.^5^^,^^19^ Although children’s mental health problems tend to cluster among children from low-socio-economic-status families, a sizable number of cases arise from middle-class families, as these comprise a greater proportion of the population.^5^ Therefore, middle-class families are major contributors to the prevalence of emotional and behavioral problems.

In Japan, little is known about the prevalence of mental health problems; consequently, which kind of interventions should be put in place remains unclear. Information is needed about how nationwide prevalence data are summarized in other countries, what kinds of measures are taken to prevent mental health problems, and whether such measures are effective or not.

Therefore, to clarify the methods that may enable implementation of an effective approach in Japan to improve child mental health at the community level, we conducted a literature review to evaluate worldwide experiences of assessing prevalence of mental health problems among children and population interventions that aimed to lower the prevalence of these problems.

Although there are already a number of review studies about prevalence rates of mental health problems among children,^20^^,^^21^ we conducted the present review so that the results could be used as baseline data for developing new interventions. While review studies of randomized controlled trials of family behavior interventions have also been reported,^22^^,^^23^ we focused on intervention at the population level.

## METHODS

Search engines and formulae used to identify relevant literature are shown in Table 1. Search results and evaluation of the identified studies are shown in Figure. We searched PubMed (a search engine provided by the United States National Library of Medicine), ProQuest (a cross-sectional search among the ProQuest Public Health, ERIC, PILOTS, Social Service Abstract, and Sociological Abstract databases), CINAHL with Full Text (a full-text database covering 17 fields concerning nursing science), and MEDLINE with Full Text (a comprehensive full-text database of medical journals) for literature published after 1980. Separate searches were conducted for prevalence data and intervention effectiveness data.

**Figure.**
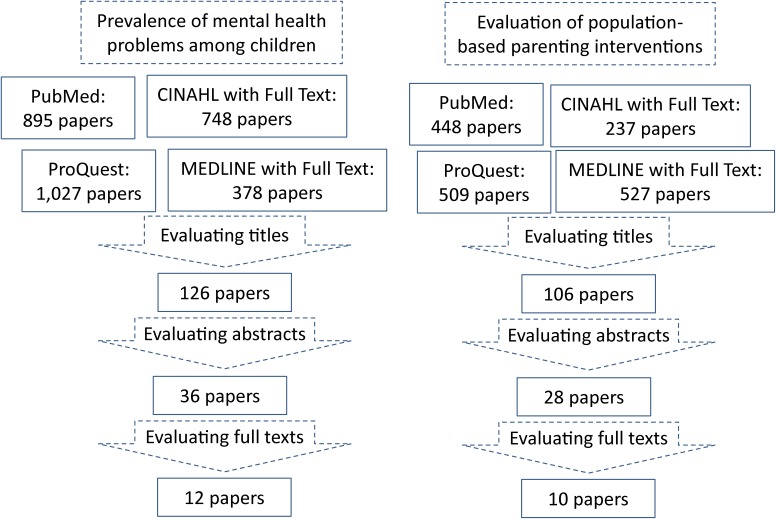
Search results and evaluation.

**Table 1.** Search strategies

Search engines	Prevalence of mental health problems among children	Evaluation of population-based parenting interventions
PubMed	“mental”[All Fields] AND “health”[All Fields] AND problem[All Fields] AND “child”[All Fields] AND (“epidemiology”[All Fields] OR “prevalence”[All Fields])	(“prevention”[All Fields]) AND (“parenting”[All Fields]) AND (“population”[All Fields])

ProQuest	((SU.exact(“MENTAL HEALTH”)) AND SU.exact(“EPIDEMIOLOGY”)) AND child	((SU.exact(“PARENTS”) OR SU.exact(“PARENTING”)) AND SU.exact(“COMMUNITY”))

CINAHL with Full Text	mental health AND children AND prevalence AND survey	parenting AND prevention AND community

MEDLINE with Full Text	mental health AND children AND prevalence AND survey	parenting AND prevention AND community

We reviewed article titles and deleted papers dealing with issues obviously different from the aims of our study. We then evaluated abstracts and identified 36 papers that dealt with prevalence of mental health problems among children using national surveys or an equivalently wide area. Through full-text evaluation, we identified 12 papers in which the prevalence of mental health problems was assessed by either the Strength and Difficulty Questionnaire (SDQ)^24^ or Child Behavior Checklist (CBCL).^25^

For evaluation of population-based parenting interventions, we identified 28 papers through abstract analysis that dealt with evaluation of interventions at a population level. Through full-text evaluation, we identified 10 papers in which the target of the intervention was child behavioral problems and the results of evaluation were reported (not the study profile). In addition to these, we conducted extra searches through PubMed for Japanese literature dealing with prevalence of behavioral problems using nationally representative data, through which we found one Japanese study. We then examined the contents of the papers to summarize the information according to the aims of our study.

## RESULTS AND DISCUSSION

### Prevalence of child mental health problems

Table 2 shows the methods and results of regional or national mental health surveys among children.^6^^,^^26^^–^^37^ In all of the included surveys, sampling was carefully done to confirm representativeness and ensure that these studies would be informative for governments planning to conduct such surveys in the future. In addition to the SDQ and CBCL, various other evaluation scales were used, such as the Center for Epidemiological Studies Depression Scale for Children,^38^ the Screen for Child Anxiety Related Disorders,^39^ and the Symptom Checklist for Attention Deficit Hyperactivity Disorders.^40^^,^^41^

**Table 2.** Survey for prevalence of mental health problem among children

Study area Country (Reference number)	Age of children	Sample size	Year period	Sampling	Evaluation scale	Selected results	Referred to specialists
Metropolitan Adelaide Australia (26)	School age (10–15 years)	Initial sample: 358 aged 10–11 years, 338 aged 14–15 years Response rate: 77% 10–11 years, 71% 14–15 years	1987	Multi-sampling stratification across schools	CBCL total difficulty	13.9 boys highest SES school 2.3 girls lowest SES school	

Child and Adolescent Component of the National Survey of Mental Health and Well-Being Australia (27)	4–17 years	4509 parents Response rate: 86%	2000	Cluster sampling of 450 censes area	CBCL wide band total Parent version of Diagnostic Interview Schedule for Children	CBCL wideband total clinical level: 14%	25%

Longitudinal Study of Children in Australia (LSAC) (6)	4–5 years	4983 children Response rate: 59%	Born between 1999.1 and 2000.2	Random cluster using two-step sampling of zip codes registered in the Medicare Australia database	SDQ 3–4 y version CES-DC SCARED FBB-HKS/ADHD CBCL	SDQ borderline total difficulty: 9.3% (0.3%–10.4%) abnormal levels: 10.5% (9.2%–11.8%)	

Bella study: mental health module for the German Health Interview Examination Survey of Children and Adolescents (KiGGS) (28)	7–17 years	2863 children and adolescents	2003–2006	Cross-sectional sub-sample of KiGGS	Extended version of SDQ FBB-HKS/ADHD	Impaired or abnormal SDQ scores: 14.5%	

BELLA study: mental health module for the German Health Interview Examination Survey of Children and Adolescents (KiGGS) (29)	11 years or older	Initial sample: 17 641 children and adolescents 14 478 children and adolescents responded	2003–2006	167 sample points participating in BELLA	SDQ	Total difficulty score abnormal or borderline: 18%	

South Italy (30)	8–9 years	3072 parents Response rate: 70%	2005	19 primary schools who agreed to participate	CBCL	Total problem parents’ report: 14.9% teachers’ report: 8.7%	

Turkey (31)	2–3 years	673 households Response rate: 95%	1996–1997	Self-weighed multistage stratified and cluster sampling	CBCL Household Questionnaire	Total problem clinically: 11.9% borderline: 18.6%	

Leipzig Germany (32)	5–6 years	3690 children Response rate: 74.3%	2009–2010	Local authority routine medical screening assessment	SDQ	Total difficulty abnormal or borderline: 16.0%	

City of Yamtai, eastern China (33)	12–17 years	1600 students Response rate: 92.3%	2010	Two-stage sampling 16 junior high-school students	CBCL Family Assessment Device	Total problem: 10.5%	

Liaong Province, northeastern China (34)	11–18 years	6205 students Response rate: 84%	2009	Two-stage sampling 30 public schools	SDQ	10.7% above cut-off for emotional and behavior problems	

United States (35)	1–3 years	1117	2008–2009	NSCAW II national representative sample (total 5872 children aged 0–17.5 years) Two-stage stratified sampling	BITESTA (screening tool for identify children at risk) CBCL	Above CBCL clinical cut-off: 10.0%	2.2% mental health service 19.2% mental health/parent training service

United Arab Emirates (36)	3 years	726 households with children of 3 years Response rate: 95.6%	2000	Multistage stratified-clustered representative sample of 2000 UAE national households	CBCL/2–3	Above CBCL clinical cut-off: 10.5%	

Japan (37)	3–15 years	Nursery schools 4135 Response rate: 44.8%	2005–2006	Randomly sampled nursery schools, elementary schools, and junior high schools	Teacher-reported mental problems that needed medical consultation	Nursery schools: 4.6% Elementary schools: 2.9% Junior high schools: 4.2%	15.9% 12.3% 12.3%
		Elementary schools 4495 Response rate: 54.7%					
		Junior high schools 2047 Response rate: 57.9%					

Among these studies, the proportions of children with clinically meaningful total difficulty according to the CBCL ranged from 10% to 20%, and the sum of internalizing and externalizing disorders was similar. The corresponding proportions of children with clinically meaningful total difficulty according to the SDQ also ranged from 10% to 20%. Behavioral problems are likely to lead to secondary mental health problems, such as depression, so managing the behavioral problems of children should be a policy priority. Only a minority (approximately 25%) of children with behavioral problems were referred to medical services in the examined studies, suggesting that the majority of children are left untreated.

These results suggest that community interventions should focus not only on high-risk populations, as is often suggested in the literature, but also on implementation as early as possible.

### Evaluation studies of population parenting interventions

Population-level interventions are potentially more effective than individual or selected approaches.^15^ Table 3 shows evaluation studies of population-level family interventions. Evaluation focused not only on behavioral problems of children but also on parental sense of confidence and parental stress or depression.

**Table 3.** Evaluation of population-level parenting interventions

Study area Country (Reference number)	Name of program	Age of target children	Years of intervention	Method of intervention	Allocation	Method of evaluation	Scales used for evaluation	Results of evaluation
Socio-economically deprived region of Eastern Metropolitan Health Region Western Australia (42)	Behavioral family intervention	2–16 years	around 2000	Large-scale population-level intervention utilizing basic health services implementation through existing services	Two quasi-experimental groups intervention: *n* = 804 control: *n* = 806	Baseline Immediately post 1-year follow-up 2-year follow-up	ECBI PS DASS	Effect size, immediately post, 1-year follow-up, and 2-year follow-up ECBI: 0.83 → 0.41 → 0.47 DASS: 0.38 → 0.29 → 0.23 PS: 1.08 → 0.59 → 0.56

Brisbane (17)	Triple P	4-to-7-year-old children	2003–2007	All five levels of Positive Parenting Program (Triple P)	10 designated areas in Brisbane 10 socio-economically matched comparison areas from Sydney and Melbourne	Computer-supported telephone interview of randomly selected families (*n* = 3000) in each area	SDQ DASS	Parental depression % above clinical level, pre-post Intervention areas: 26.7% → 19.7% Control areas: 19.1% → 18.6%
						Baseline 2-year post-intervention		Total difficulty % above clinical level, pre-post Intervention areas: 13.9% → 10.9% Control areas: 9.7% → 10.4%

Canada (8)	Parenting program Solving discussion Role play Modelling Homework	Junior kindergartners	Around 1990	Randomly assigned to (1) 12-week individual, (2) 12-week large-group, or (3) waiting-list	2564 families above the 90th percentile on the risk scale randomized to large-group, community based parent-training program or clinic-based, individually delivered parent-training program	Baseline Post-intervention	CBCL SOFC 1-hour home observation	Greater improvements in behavior problems at home in Community/Group intervention and better maintenance Significant time effect in CBCL, POFC, home observation (MANOVA)

South Carolina United States (43)	Triple P	Under 8 years	2006–2008	2 years of intervention with all 5 level of Triple P system by 649 service providers	Random allocation of 18 counties in a southeastern state of the United States	Baseline 2-year post-intervention	Rate of substantial CM Out-of-home placement Hospitalization or emergency visit from CM	Reduction in rates, effect size substantial CM: 1.09 out-of-home placement: 1.02 hospitalization or emergency room visit: 1.14

England (44)	Triple P Incredible Years (school version) Strengthening Families Strengthening Community (SFSC)	8–13 years	2008	Families with children at risk of antisocial behavior assigned to one of three parenting programs	Random allocation of local authorities (LAs) to three programs (6 LAs for each) Incredible Years: 56 groups Triple P: 142 groups SFSC: 68 groups	Numbers of families evaluated	WEMWBS PS Adolescent PSOC	Effect sizes, ranges across programs WEMWES: 0.44–0.88 PS: 0.57–0.77 PSOC: 0.33–0.77 SDQ: −0.47–−0.71 SFSC: less effect than other two programs
						Pre-course data Incredible Years: 473 Triple P: 1084 SFSC: 650		
						Post-course data Incredible Years: 240 Triple P: 515 SFSC: 366		

England (45)	Triple P Strengthening Families Strengthening Community (SFSC) Incredible Years Strengthening Families Program (SFP) Families and Schools Together (FAST)	8–13 years	2009–2011	Delivery through usual health services	Intervention LAs within 47 LAs representative of 147 LAs, 43 LAs which could collect data LAs are free to select any one or more of five programs	Pre-intervention Total: 6143 Triple P: 3171 SFSC: 868 Incredible years: 782 SFP: 969 FAST: 104	WEMWBS PS Adolescent	larger effect in Triple p but no significant differences among programs combined effect size parenting laxness 0.72 (PS) over activity 0.85 (PS) parent well being 0.79 (WEMWEB) conduct problems 0.45 (SDQ)
						Post-intervention total: 3325 one hour after: 1035		

Inner city of Kingston Jamaica (46)	Incredible Years Teacher Training	3–6 years	2009–2010	Training all the teachers Mentors in class Workshops	Cluster randomization 24 out of 50 community pre-schools: Intervention (*n* = 12) vs control (*n* = 12)	Baseline Post-intervention	1-hour home observation ECBI SDQ School attendance	Effect size reduced conduct problems: 0.42 (observation) increased friendship skills: 0.74 (observation) reduction in behavior difficulties, teacher report: 0.47 reduction in behavior difficulties, parent report: 0.22 increased Social skill, teacher report: 0.59 increased Child attendance: 0.30
					Three children from each class with highest level of teacher-recognized conduct problems (225 children)			

Socially deprived area in London England (47)	Empowering Parents Empowering Communities	2–11 years	2010	Trained and accredited peer facilitator	116 help-seeking families allocated to intervention (*n* = 59) or waitlist (*n* = 57)	Baseline Post-intervention	ECBI SDQ PS PSI	Effect size ECBI intensity: 0.37 SDQ total difficulty: 0.28 Parenting scale: 0.80 Parenting stress: 0.24

Suburban Oslo Norway (48)	Early Intervention for Children at risk for Development Behavior Problems (EICR)	6–12 years	2004–2005	Module based training of local professionals	7 elementary schools 271 teachers Quasi-experimental pre-post design Randomly selected intervention and control areas All identified children in intervention area	Baseline Post-intervention	Staff-reported problem incidence in classroom	Significant intervention effect F(1215) = 11.69 No significant time effect

Ireland (49)	6-week prevention version of the Parents Plus Early Years Programme (pilot study)	3–12 years	around 2008	6-week intervention Trained facilitator of community professionals	Nationwide recruitment through routine school activities or family support services 40 parents attended 29 parents completed evaluation	Baseline Post-intervention	SDQ CPG WSRF	Effect size SDQ total difficulty: 1.65

ECBI, Eyberg Child Behavior Inventory; PS, Parenting Style; DASS, Depression, Anxiety and Stress Scales; SDQ, Strength and Difficulty Questionnaire; CBCL, Child Behavior Checklist; SOFC, Sense of Family Coherence; CM, child maltreatment; WEMWBS, Warwick-Edinburgh Mental Well-being Scale; PSOC, Parenting Sense of Competence Scale; PSI, Parenting Stress Index; CPG, client defined problems and goals; WSRF, weekly session rating form.

The majority of these studies^8^^,^^17^^,^^42^^–^^49^ recruited intervention samples using population-based sampling strategies to recruit high-risk children, implemented parenting programs, and evaluated the effectiveness among samples through pre- and post-intervention assessments. Among such studies, only half also included control groups for comparison.

Two studies used variant types of study design, in which the intervention was provided at various levels of intensity, and families received the relevant intensity of intervention based on the degree of behavioral issues.^17^^,^^43^ Through such an approach, almost all families in the study area receive some kind of intervention. One of the two studies evaluated the effect using a questionnaire sent to randomly sampled families within the region.^17^ The other study measured the occurrence of child maltreatment before and after 2 years of intervention, which corresponds to a long-term effect.^43^ Although the assessed outcome was not child behavior or family well-being, child maltreatment tends to occur through severe impairment of such indicators. In reports from Jamaica^46^ and Norway,^48^ the intervention was conducted by teachers, which is a variant type of parenting intervention.

Although most of these studies summarize the results using effect sizes, they also report decreases in the percentages of children with assessment scores above the clinical level. Improving the outcomes for high-risk children can lead to considerable reductions in the proportions of children with problems at the population level of each scale. Some studies^44^^,^^45^ implemented different programs among communities, with the effectiveness compared among programs. Each program had some degree of prevalence change in each setting.

Through our review, we found that researchers are still seeking better methods for community intervention and evaluation. The most important goal of a given method is to deliver programs to all families in the community who need support.^15^

Table 4 provides a comparison among manualized parenting programs (ie, those with manuals, textbooks, or other published materials)^50^^–^^53^ that were implemented and evaluated through a population approach.^17^^,^^43^^–^^46^ Almost all of the programs were based on scientific theories, were disseminated among many countries, and introduced a universal approach targeting all families in a community. Each program also had unique characteristics that distinguished it from the others. The Positive Parenting Program (Triple P)^50^ provides a multilevel approach according to the severity of the problem. Optional interventions were provided according to risk level in Strategic Prevention Framework programs.^53^ Incredible Years^52^ provided not only parent versions but also child and teacher versions. One of the programs^51^ was culturally sensitive and designed for disadvantaged families. The variety of available programs allows policymakers to choose the program that is suitable for the problems of their own communities.

**Table 4.** Parenting programs implemented using a population approach

Name of program	Theoretical basis	Characteristics	Target	Developer	Dissemination
Triple P	Child development Therapeutic practice Social learning	Five levels suitable for each level of problem	Every parent of children under 16 years	Matthew R. Sanders University of Queensland Australia	25 countries

Strengthening Families Strengthening Communities (SFSC)	Family stress Children’s development Social learning Ecological	Culturally sensitive program	Any families including ethnic minority children	Race Equity Foundation United Kingdom (formerly developed in United States)	United Kingdom United States

Incredible Years	Social learning Self-efficacy Cognitive behavioral Piaget’s developmental	Parent version Child version Teacher version	Children at risk	Carolyn Webster-Stratton University of Washington United States	26 countries

Strengthening Families Program (SFP)	Biosychosocial Vulnerability Resiliency Family process	Optional interventions according to level of risks and age of children	Caregivers of any children aged 6–17	Karol Kumpfer Office of drug control University of Iowa United States	26 countries

Systematic screening of preschoolers or schoolchildren may identify issues that can be considered precursors to later problems, which suggests that universal screening may be beneficial.^7^ An approach that utilizes a universal service system that is accessed by all or nearly all children and an acceptable screening tool for the systematic identification of at-risk children are needed.

Population exposure to interventions may result in a significant reduction of the total number of behavioral problems, even though reductions at the individual level may be modest.^17^ Children with mild behavioral problems make up a large part of the community population, and their improvement could be of substantial benefit to the community.^17^

### Future perspectives

We reviewed the prevalence of mental health problems among children in the community and the effect of universal family intervention at the population level, which may reduce the prevalence of children’s mental health problems. The reviewed evidence shows that children’s and families’ mental health improved on a variety of measures as a result of community intervention. In particular, a decrease in the prevalence of child maltreatment was reported through the community approach.

If we were to choose the kind of intervention method likely to the have greatest benefit for the population, it would be a comprehensive intervention, such as Triple P, which targets not only severe cases but also apparently normal children showing precursors to later problems. In Japan, construction of a surveillance system of mental health problems among children would be helpful to guide policymaking for community interventions and to evaluate the effectiveness of such interventions.

One of the most important aims of community intervention is to modify mild behavioral problems to prevent the future development of more serious mental problems. Therefore, large-scale, long-term follow-up studies will be needed to evaluate the effect of these preventive measures.

### Limitations and conclusions

There are some limitations in the present study. First, the issues identified in the present study are not covered by only medical or health science. Our approach was limited to health and medical databases, but a more extensive search through social, psychological, and cultural databases may identify other relevant research. However, sufficient evidence was obtained from the databases to which we had access to obtain a general overview of key issues. Second, there is publication bias in the literature, as negative results are not likely to be published. To minimize publication bias, better search methods or methods of analysis are needed to identify positive and negative results. Despite these limitations, the information presented here could be useful for political decision-making regarding the conduct of mental health surveys among children and the delivery of adequate community-based interventions.

In conclusion, we clarified through a literature review that mental health problems among children are common across countries and require political commitment to address issues at a population level. We identified several promising community-level family interventions that may be effective in addressing such problems.

## ONLINE ONLY MATERIAL

Abstract in Japanese.
