# Supplementary material for: Prevalence of Children’s Mental Health Problems and the Effectiveness of Population-Level Family Interventions
Source: J Epidemiol. 2015 Aug 5;25(8):507–16. doi: 10.2188/jea.JE20140198 (PMC4517988; doi:10.2188/jea.JE20140198)
Supplement: Abstract in Japanese. [file je-25-507-s001.pdf]

# 小児のメンタルヘルス有病割合と地域レベルでの親支援介入のもたらす効果

加藤則子[1]　柳川敏彦[2]　藤原武男[3]　アリーナ・モラウスカ[4]

1 国立保健医療科学院　地域保健システム研究分野　(現十文字学園女子大学)
2 和歌山県立医科大学　保健看護学部
3 国立成育医療研究センター　社会医学研究部
4 クイーンスランド大学　家族子育て支援研究所

小児及び思春期におけるメンタルヘルス問題の有病割合は極めて重要性が増している指標である。子どものメンタルヘルスに早くから介入することは、子どもがより年齢が大きくなってからの介入より有効である。地域レベルでの予防活動においては、健康課題の有病割合を把握した上で、必要なレベルのサービスを提供する必要がある。小児のメンタルヘルス有病割合は多くの国で明らかにされている。予防活動においては、環境を最善にすることが強調されている。親は子供の成長に大きな影響を及ぼすことから、育児技術を向上させるための親教育に大きな関心が注がれている。そのなかで、親支援介入の地域レベルでの効果を明らかにする公衆衛生的アプローチが求められる。本文献レビューによって、小児メンタルヘルスの有病割合が国レベルで把握されている事例が多く、地域レベルでの親支援介入が地域での小児メンタルヘルス問題の有病割合を減少させうることが明らかになった。

小児　メンタルヘルス　有病割合　親支援介入　地域レベル
